# Beyond the germ: Rethinking implant-related infections as a host–microbiota–biomaterial ecosystem

**DOI:** 10.1016/j.bioflm.2026.100352

**Published:** 2026-02-06

**Authors:** D.C. Coraça-Huber, C. Spiegel, B.F. Moraes, R. Arora

**Affiliations:** a Research Laboratory for Biofilms and Implant Associated Infections (BIOFILM LAB), Department of Orthopaedics and Traumatology, Medical University of Innsbruck, Müllerstraße 44, 6020, Innsbruck, Austria; b Departmento de Odontologia Restauradora, Faculdade de Odontologia de Piracicaba, Universidade Estadual de Campinas, Avenida Limeira 901, CEP: 13414-903, Piracicaba, SP, Brazil

**Keywords:** Implant-related infection, Biofilm, Host–microbiota–biomaterial interactions, Oxidative stress, Detoxification, Colonization resistance, Systems biology, Germ theory, Terrain theory

## Abstract

Implant-related infections remain one of the most challenging complications in modern medicine, often leading to implant failure, revision surgery, significant patient morbidity and high rate of patient mortality. Traditionally framed within the germ theory paradigm, their pathogenesis has been attributed primarily to microbial contamination and biofilm formation. However, growing evidence reveals a far more complex picture in which infection susceptibility emerges from the dynamic interplay between **host biology, microbiota composition, biomaterial properties, and environmental influences.** Host immune tone, metabolic status, and systemic exposures shape the tissue environment in ways that either resist or facilitate microbial colonization. The microbiota contributes to this balance not only by mediating immune responses and providing colonization resistance but also through metabolites and detoxification mechanisms that influence local and systemic immunity. Biomaterials themselves are active participants in this ecosystem: metal ion release, corrosion, and surface properties modulate both host responses and microbial behavior.

A particularly intriguing concept is the potential **dual role of biofilms** — not only as pathogenic reservoirs but also as **detoxification systems** that sequester metal ions and buffer oxidative stress at the host–implant interface. Recognizing this duality reframes biofilms as biological structures that may, in part, participate in protective containment, albeit at the cost of sustaining low-grade inflammation and risk of septicemia.

Looking forward, the integration of **systems biology approaches** promises to transform our understanding and management of implant-associated infections. Multi-omics profiling, predictive modeling, engineered probiotics, dietary modulation, and novel implant surface designs represent emerging strategies that target the entire host–microbe–material interface. By bridging insights from immunology, microbiome science, biomaterials research, and digital technologies, a new paradigm is emerging — one that views infection prevention and treatment not simply as microbial eradication, but as the cultivation of a resilient biological and material ecosystem around the implant.

## 1 Introduction

Infection remains a major challenge in modern biomedical practice, particularly in the context of surgical wounds and implant-related procedures. The increasing number of joint replacements, dental implants, and cardiovascular devices has been accompanied by a parallel rise in chronic, biofilm-associated infections that are notoriously difficult to treat and often require revision surgeries [1]. Conventional explanations for these complications have traditionally been rooted in the so called, *germ theory*, emphasizing microbial exposure, pathogen virulence, and contamination risk. However, there is a longstanding and resurging debate around the *terrain theory*, which posits that the host's internal environment—encompassing immune tone, oxidative stress, tissue perfusion, and microbiota balance—plays an equally central role in determining whether microbial exposure leads to infection [[2], [3], [4]]. Recent advances in infection science suggest that neither theory alone is sufficient. Instead, it is the interplay between microbial challenges and the host “terrain” that dictates outcomes. Metallic implants, for example, are not inert; wear, corrosion, and local biochemical processes release ions and nanoparticles such as titanium, cobalt, or nickel, which can trigger oxidative stress, tissue damage, and chronic inflammation [5]. Simultaneously, the human microbiota—both local (skin, oral, vaginal) and systemic (gut)—acts as a determinant of infection susceptibility, with dysbiosis predisposing to poor wound healing and colonization by opportunistic pathogens [6]. The immune system, shaped by both, biomaterial debris, and microbiota, emerges as a key mediator, capable of either containing pathogens or fostering a permissive environment for biofilm persistence [7]. This review aims to synthesize current knowledge on these interconnected dimensions, bringing together insights from immunotoxicology, microbiome science, and biomaterials research. By bridging germ theory and terrain theory, we seek to outline how environmental and lifestyle factors, biomaterial properties, and host–microbe–metal interactions converge to shape infection susceptibility (see Table 1).

**Table 1:** Future directions, key insights and innovation opportunities.

Domain	Current Understanding	Innovative Research Directions	Potential Clinical Applications
**Host Factors & Immune Modulation**	Infection susceptibility depends on immune tone, genetics, comorbidities, and systemic inflammation.	- Develop predictive “infectogenomic” profiling for infection risk. - Explore immunomodulatory therapies targeting chronic inflammation around implants. - Investigate the impact of immune ontogeny and aging on implant outcomes.	- Personalized preoperative risk assessments. - Tailored immunonutrition and anti-inflammatory interventions before surgery.
**Microbiota & Colonization Resistance**	Microbiota influences immune maturation, pathogen exclusion, and wound healing. Dysbiosis increases infection risk.	- Design microbiota-based biomarkers to predict infection risk. - Develop targeted probiotic or symbiotic therapies to shape peri-implant microbiota. - Study microbiota–vaccine interactions in implant patients.	- Oral or local probiotic therapies. - Microbiota transplantation to restore colonization resistance. - Modulation of gut–bone–skin axes to improve implant outcomes.
**Diet, Lifestyle & Environment**	Diet, smoking, pollutants, and micronutrient status influence immune competence and infection outcomes.	- Elucidate how dietary metabolites modulate immune tone and microbiota composition relevant to implants. - Longitudinal studies linking environmental metal exposure to implant infection risk.	- Pre-habilitation programs optimizing diet and micronutrients. - Lifestyle modification protocols integrated into surgical planning.
**Biomaterials & Metal Interactions**	Metals released from implants trigger oxidative stress, inflammation, and microbial adaptation.	- Design corrosion-resistant alloys and ceramic alternatives. - Engineer surfaces with antioxidant coatings or controlled ion release. - Explore effects of released ions on microbiota composition and immune activation.	- Biomaterials with lower ion release. - Surfaces that reduce oxidative stress and inflammation. - Patient-specific implant material selection.
**Biofilms & Detoxification**	Biofilms are classically seen as pathogenic, but EPS and microbial mechanisms may sequester metals and buffer oxidative stress.	- Quantify detoxification potential of biofilms in vivo. - Engineer biofilm-mimicking coatings that chelate metals without enabling pathogenic persistence. - Identify genetic determinants of biofilm detox functions.	- Therapeutic strategies separating beneficial sequestration from infection risk. - Adjunctive therapies modulating biofilm behavior instead of solely eradicating it.
**Microbes as Therapeutics**	Certain commensals promote wound healing, modulate inflammation, and detoxify metals.	- Engineer probiotic strains to express metal-binding EPS or redox enzymes. - Develop microbial consortia designed for implant protection and tissue regeneration.	- Coating implants with beneficial biofilms. - Probiotic-based wound dressings or local therapies for contaminated wounds.
**Systems Biology & Predictive Models**	Infection emerges from complex host–microbe–material interactions.	- Integrate multi-omics (microbiome, metabolome, immunome) into predictive models. - Build digital twins of host–implant ecosystems to simulate infection scenarios.	- Precision medicine platforms predicting patient-specific infection risk. - Decision-support tools for personalized implant strategies.
**Artificial Intelligence and Digital Tools**	Current approaches to PJI prevention and management rely mainly on clinical risk factors, standard microbiological methods, and empirical decision-making, often without integrating complex host, microbial, and biomaterial data.	AI can integrate multi-omics, clinical, and imaging data to build predictive models of infection risk, guide patient-specific prevention strategies, and support decision-making. Machine learning can accelerate biomaterial design, improve biofilm detection, and enable digital twins that simulate host–microbe–implant dynamics.	Personalized risk stratification before surgery, early detection of dysbiosis or biofilm formation, optimized antibiotic selection, tailored surgical planning, and faster development of next-generation implant surfaces.

## 2 Methods

This review was conducted as a structured narrative synthesis of the current literature on host–microbiota–biomaterial interactions and their role in implant-related infections. We performed a comprehensive literature search using PubMed, Web of Science, and Scopus databases. The search strategy combined terms related to implant-associated infections, biomaterials, metals, microbiota, immune modulation, and host susceptibility. All references were imported into Covidence (Veritas Health Innovation, Melbourne, Australia) for systematic screening and organization. Titles and abstracts were independently reviewed by the authors to assess relevance based on predefined inclusion criteria: (i) original research or review articles addressing infection susceptibility in the context of implants or surgical interventions; (ii) studies examining host immune factors, microbiota composition, or biomaterial properties; and (iii) publications reporting interactions between metals or biomaterials and microbial or host responses. Full texts of eligible articles were then retrieved and assessed for final inclusion. Additional references were identified through manual searches of the reference lists of key articles and recent review papers. Discrepancies in study selection were resolved through discussion among the authors. The included literature was synthesized thematically, structured around four main domains: (1) host factors and infection susceptibility, (2) environmental and lifestyle influences, (3) biomaterial–microbe interactions, and (4) integrative approaches for infection prevention.

## 3 Theoretical and historical framework

### 3.1 Terrain theory and infection prevention

Louis Pasteur established the concept that certain microorganisms were directly responsible for causing diseases, which became known as the germ theory. On the other hand, Claude Bernard and Antoine Béchamp proposed a different view, centered on the role of the organism itself, or “terrain”, as a key element in the origin of diseases, according to this line of thought, a body in balance would not offer the necessary conditions for external microorganisms to develop pathogenic activity [8]. In recent years, the proposal for an integrative approach between germ and biological terrain theories has been strengthened, known as germ-terrain duality, which considers that the development of a clinical condition depends not only on the presence of an infectious agent, but also on the physiological and immunological state of the host. In the case of the microbiota, this idea becomes very relevant. Commensal microorganisms, for example, can act both in a beneficial and pathogenic way, depending on the local balance and the individual immune response [9]. Scott et al. (2020) reinforce this perspective by showing that infection prevention is not necessarily linked to the widespread elimination of microorganisms, but rather to the real reduction of transmission risks, without compromising the integrity of the microbiota [10].

### 3.2 Endogenous microorganisms and disease resistance

The endogenous microbiota plays a fundamental role in defending against infections and regulating host immunity. From the earliest moments of life, commensal microorganisms contribute to the maturation of the immune system, in addition to favoring the establishment of physical and biochemical barriers that hinder colonization by pathogens [11,12]. Previous exposure to pathogens appears to influence the dynamics of the microbiota, favoring a more efficient response to the occurrence of new episodes. This adaption process appears to be associated with an increase in the metabolic activity of commensal microorganisms, with greater production of antimicrobial compounds [13]. Still described by Stacy et al. (2022), who used an animal model to demonstrate that prior contamination by pathogens can lead to increased resistance to infection, it was observed that even in the absence of apparent systemic changes, microorganisms were able to selectively inhibit the respiration of pathogens, promoting resistance to colonization [13]. The ability to influence resistance through a microbiota previously adapted to infections suggests that the interaction between microorganisms and the immune system goes beyond coexistence. In this sense, the use of probiotics or microbial components has been studied as a way to rebalance the immune system, especially in cases of dysregulation or chronic inflammation [12]. Imbalances in the composition of the microbiota, caused by inadequate diets, repeated infections or excessive use of antibiotics, can impair the body's ability to maintain effective immune responses, increasing the risk of infections, chronic inflammation and adverse clinical problems [14].

### 3.3 Host factors in infection susceptibility

An individual's ability to contract an infection is not solely linked to the characteristics of the pathogen, but is also related to elements inherent to the organism, such as genetic diversity, the condition of the immune system, age, resident microbiota and environmental influences [15]. The explanation given for why certain hosts develop severe forms of the disease and others present mild or even asymptomatic manifestations is related to the different immunological and genetic profiles [16,17]. Research indicates that genetic variations adjust the expression of genes linked to the inflammatory response, antiviral defense and the identification of invaders, directly impacting protection against infectious agents [18,19], which leads to the idea of infectogenomics, where the response to infection is determined by the host genome as a whole and the selective pressure exerted by the pathogen [18]. Age is also a factor that influences susceptibility to infections. Newborns depend on maternally provided immunity and early colonization by the microbiota, which helps build the immune system in the first weeks of life [20], while older adults become more vulnerable to infections due to conditions such as immune system aging and chronic inflammation [15,21]. Furthermore, microbial diversity and interactions between species in environments also influence susceptibility to infection. The dilution theory suggests that communities exposed to environments with a more diverse microbiota tend to have a lower incidence of infectious diseases, limiting the transmission of pathogens between susceptible hosts [22]. Therefore, an integrative approach is needed that considers both the biological aspects inherent to the host and the environmental context in which it is inserted.

### 3.4 Immune modulation by microbiota

As is already well investigated, the intestinal microbiota is a regulator of the immune system, which influences innate and adaptive immunity, which occurs through interactions between microorganisms and lymphoid tissues in the intestine, important in maintaining immunological homeostasis and controlling inflammatory responses [23]. A previous study demonstrated that microbiota metabolites, such as fatty acids, not only favor immune modulation with the differentiation of regulatory T lymphocytes, but also promote benefits to intestinal tissue, strengthening its epithelial barrier and reducing permeability, hindering the transit of pathogens to the blood stream [23]. In addition, it has been reported that the microbiota can also directly modulate the systemic immune response, interfering with the efficacy of vaccines and the cytokine production profile after immunization [24]. Imbalances or even absence of microbiota have been associated with immune dysregulation, due to reduced production of secretory IgA, increased systemic inflammatory responses and decreased response to vaccine antigens. This relationship between microbiota and immunity indicates that interventions that preserve or help restore intestinal microbial composition can be considered promising strategies to improve the immune response in different therapeutic or even preventive contexts [24].

### 3.5 Diet and infection susceptibility

Diet is directly related to susceptibility to infections, interfering with both the immune system and the composition of the intestinal microbiota [25]. Western diets that are rich in refined sugars, saturated fats, ultra-processed foods, and low in fiber are associated with inflammation, breakdown of the epithelial barrier, and reduced microbial diversity, favoring the establishment of an environment conducive to the translocation of pathogens and immune dysfunction [26]. A previous study using the *Drosophila melanogaster* as animal model [27], demonstrated that diets high in sugar significantly increase susceptibility to bacterial infections, even in organisms considered healthy, in addition to impairing the activation of innate immunity pathways and the ability to defend against Gram-negative bacteria [27]. On the other hand, as already well established, nutritional deficiency can also impair immune capacity. Mice that were subjected to dietary and water restriction showed a marked increase in mortality after infection by resistant pathogens, such as Methicillin-resistant *Staphylococcus aureus*. The occurrence of deleterious effects on immunity, such as failure of infectious containment, accompanied by macrophage dysfunction and accumulation of renal bacterial load, was also demonstrated, but which were reversed after rehydration [28].

## 4 Environmental and lifestyle factors in infection risk

The risk of infection after implant surgery is not solely dictated by intraoperative sterility or bacterial exposure; environmental and lifestyle factors strongly influence outcomes. At the surgical level, preventive measures such as strict asepsis, optimized operating room conditions, and careful implant handling remain indispensable. Yet, growing evidence highlights the role of broader environmental exposures—including pollutants and heavy metals—that may compromise immune competence and wound healing [29]. Chronic exposure to cadmium, lead, or mercury, for example, has been associated with impaired phagocytic function and altered cytokine responses, creating a systemic background more permissive to infection [30]. Lifestyle determinants such as nutrition, smoking, alcohol consumption, and comorbidities also contribute significantly. Malnutrition and deficiencies in micronutrients such as zinc, selenium, and vitamin D are linked to impaired tissue repair and increased surgical site infection rates [[31], [32], [33]]. Conversely, nutritional optimization before surgery can improve immune resilience. Smoking and poorly controlled diabetes are well-established risk factors for implant infection, largely due to microvascular dysfunction, hypoxia, and impaired leukocyte activity [[34], [35], [36]]. Preventive strategies targeting these modifiable risk factors have shown tangible benefits.

Diet plays a pivotal role in shaping the composition and metabolic output of the gut microbiota, with significant consequences for immune function and infection susceptibility. A key mechanism underlying this link is the microbial fermentation of dietary fibers and resistant starches into ***short-chain fatty acids (SCFAs)*** — primarily acetate, propionate, and butyrate. These metabolites exert profound **anti-inflammatory and immunomodulatory effects,** influencing both local mucosal immunity and systemic immune responses. SCFAs promote the differentiation of regulatory T cells (Tregs) and suppress the production of pro-inflammatory cytokines such as IL-6 and TNF-α, thereby contributing to immune homeostasis and enhanced resistance to pathogens [11,37,38]. Moreover, dietary patterns rich in fiber and polyphenols have been shown to increase SCFA production and improve epithelial barrier function, reducing translocation of microbial products and dampening systemic inflammation [25,39]. Conversely, Western-style diets low in fermentable substrates and high in saturated fats and sugars reduce SCFA-producing taxa and are associated with increased inflammation and infection risk [28,40]. These findings highlight how **dietary modulation of the microbiome and SCFA production represents a powerful, host-centered strategy** to influence the inflammatory milieu relevant to implant integration and infection prevention.

### 4.1 Preventive strategies for implant‐related infections

Preoperative glycemic control, smoking cessation, and weight reduction reduce the incidence of prosthetic joint infection [41]. Beyond systemic health, perioperative interventions such as reducing surgical duration, avoiding thermal necrosis of bone, maintaining tissue oxygenation and soft tissue management are key determinants of infection prevention [42]. An emerging approach is the use of probiotics and microbiome modulation as preventive strategies. Several clinical studies have demonstrated that probiotics may reduce peri-implant inflammation and mucositis [43], though their effects on established peri-implantitis remain less conclusive [44]. Engineered probiotic biofilms have even been explored as implant coatings, capable of preventing colonization by pathogenic bacteria such as *Staphylococcus aureus* while simultaneously promoting osseointegration [45]. According to the last international consensus meeting on muskuloskeletal infections [46], preoperative nutritional status—particularly malnutrition, vitamin D deficiency, and gut dysbiosis—plays a significant and modifiable role in surgical site and periprosthetic joint infection risk in orthopaedic surgery, making nutritional screening and optimization essential components of prevention.

## 5 Microbiota, detoxification, and metal interactions

### 5.1 Detoxification pathways and microbiota

The intestinal microbiota plays a very important role in the detoxification of the organism, given its ability to transform toxic compounds and regulate metabolic and inflammatory pathways. It has been reported that tryptophan-derived metabolites produced by *Bifidobacterium bifidum* reduced hepatic inflammation, restored the integrity of the intestinal barrier, and even promoted protective effects in animal models with liver dysfunction [47]. Furthermore, as previously reported [48], the microbiota was also able to modulate the toxicity of an environmentally toxic compound, amygdalin, attenuating its deleterious effects through the biotransformation mechanism and the regulation of antioxidant and inflammatory genes in the liver. Thus, the microbiota appears to not only metabolize toxins, but also influence the host response, functioning as an important component in detoxification against toxic substances.

### 5.2 Microbial detoxification of heavy metals

The presence of heavy metals such as cadmium, lead, chromium and arsenic in the environment is a real risk to human health and ecosystems. Because they accumulate in tissues and cause progressive toxic effects, these elements have increasingly been the subject of scientific studies in various areas. Among the strategies to reduce their impacts, the ability of certain microorganisms to act in the detoxification of these metals has been increasingly addressed, especially due to the possibility of sustainable applications in environmental and biomedical contexts [49]. It has been reported that chronic exposure to metals can generate accumulation in phagocytic defense cells in animal models, with increased production of reactive oxygen species (ROS), DNA fragmentation and mitochondrial morphological alterations. Changes in the expression of pro and anti-inflammatory cytokines were also observed, indicating a dysregulated immune response associated with intracellular metal load [50]. Microbial bioaccumulation differs from biosorption in the mechanism by which it occurs. While biosorption is given by the passive binding of metal ions to the cell surface, bioaccumulation refers to the active uptake and intracellular retention of these elements. This process has been explored as a potential tool for bioremediation, mainly in highly contaminated environments [51,52]. More than one hundred strains of lactic acid bacteria (LAB) have been tested for their metal biosorption capacity, and all of them show positive responses through interactions between metal ions and functional groups of the cell wall, such as carboxyls and phosphates. This action can be intensified by the production of exopolysaccharides (EPS), which are extracellular structures that act as chelators, modulating bioavailability and reducing systemic toxicity of organisms exposed to heavy metals [53,54]. The production of EPS by lactic acid bacteria in the gastrointestinal environment has also been associated with the ability to form stable complexes with metal ions, as observed by Wang and collaborators [54], who identified interaction between EPS and Pb and Cd ions, reducing the absorption of these elements in the organism and promoting fecal elimination of these elements. The therapeutic potential of EPS has also been linked to additional intestinal epithelial barrier protection [55]. Furthermore, some bacteria that colonize the environment are capable of transforming highly toxic compounds such as arsenate (As^5+^) and cyanide. According to Olaya-Abril et al. (2024), bacterial reductase or cyanidase enzymes confer the ability to transform these substances into less toxic or volatile forms. In addition to these compounds and heavy metals, the intestinal microbiota also plays an important role in the biotransformation of metals such as bismuth, affecting their distribution and systemic toxicokinetics [56,57]. Reinforcing this panorama, a detailed review on recent microbial strategies applied to heavy metal bioremediation, including bacterial consortia and genetic engineering to increase affinity and adsorption efficiency in contaminated environments was also reported in a previous study [58]. Bioengineering has proposed modifying the cell surface of certain microorganisms with the aim of increasing the selectivity and capacity for intracellular accumulation of metals from heavy metal-binding peptides, increasing bacterial affinity for metal ions [52]. Furthermore, the conversion of highly toxic metallic forms into less reactive compounds by microorganisms has also been reported in contaminated wounds, which can be considered as an alternative way to mitigate the deleterious effects of these contaminants also in tissue repair processes [49].

## 6 Heavy metals, inflammation, and immune modulation

Aluminum and nickel, though often present at low concentrations in processed food, industrial materials, and even personal care products, have been shown to interfere with immune activity. Some studies suggest they can promote inflammation, suppression of adaptive pathways, effects that seem to depend not only on dosage, but also on how the exposure happens and which cells are involved [59,60]. Aluminum have been part of vaccine formulations for decades, mainly due to their ability to trigger innate immune responses. What seems to drive this effect is the release of mitochondrial DNA and activation of the inflammasome, a pathway that ultimately promotes local inflammation. Some reports also point to aluminum accumulation in tissues as a contributing factor, possibly extending antigen exposure and maintaining an inflammatory tone in the surrounding environment [59]. In contrast, nickel has been associated with immunosuppressive effects. In some studies, macrophages exposed to low concentrations of this metal showed reduced secretion of pro-inflammatory interleukins and TNF-α. However, this impact was intensified in the presence of cadmium, indicating that combined exposure to certain metals may exacerbate damage to cellular solutions and basic immune function [60]. Furthermore, exposure to metallic compositions containing nickel, aluminum, cadmium and other elements has been linked to mitochondrial oxidative stress and systemic inflammation, affecting pathways such as MAPK and NF-κB, and also altering circulating cytokines [61]. Additionally, a study with human liver cells reported that metallic compounds simultaneously activate cell death pathways and chronic inflammatory responses, showing that heavy metals have toxic potential [62].

### 6.1 Heavy metals and chronic inflammation

Prolonged exposure to heavy metals can trigger inflammatory mechanisms, promoting the emergence of chronic problems. This occurs through oxidative stress, activation of the inflammasome and changes in mitochondrial function, which keep inflammatory signals active even without infection or apparent injury [59,61,63]. Furthermore, both zinc deficiency and excess appear to unbalance the immune system. Low levels generally increase oxidative stress, pro-inflammatory cytokines and activate NF-κB. Excess zinc, in turn, can also cause inflammation, showing that proper regulation of this metal is crucial. In addition, zinc modulates T-cell responses, which are essential for controlling inflammation [64]. It is concluded that deficient or exaggerated exposure to metals impairs the body's ability to regulate inflammation, contributing to the persistence of inflammatory responses and even the development of chronic diseases.

### 6.2 Heavy metal toxicity and tissue healing

Tissue healing is a dynamic process, with inflammatory, proliferative and remodeling phases, which heavy metals can impair, both due to their immunosuppressive effects and due to changes in the cellular signaling involved in repair [65,66]. A previous study [65] revealed that chronic exposure to low doses of CdCl_2_ in mice significantly impaired skin healing, with reduced neutrophil infiltration, expression of chemokines (CXCL1 and CXCL2), and pro-inflammatory cytokines (TNF-α, IL-1β, and IL-6). The authors linked these effects to inhibition of the ERK1/2 and NF-κB pathways, which are crucial in the initial inflammatory response; this imbalance impairs the early phase of healing and, consequently, the entire repair process [65,66]. In another study [66], it was observed that rats exposed to topical cadmium presented persistent edema, prolonged inflammatory infiltration and abnormal epithelial growth in skin lesions, indicating that local and systemic exposure to the metal may impair the inflammatory resolution necessary for adequate healing progression. On the other hand, there are studies that suggest that some metal ions, such as zinc, copper and manganese, in appropriate compositions, can promote healing, especially of diabetic wounds, acting as enzymatic cofactors or signaling agents for cell proliferation and angiogenesis [62].

### 6.3 Endogenous defense mechanisms and metal toxicity

Our bodies have several defense tactics to reduce the damage caused by exposure to heavy metals. Antioxidant systems, proteins that bind to metals, and cellular repair processes work in different ways to regulate oxidative stress, restrict the presence of metals, and help maintain tissue integrity [67]. Heavy metals stimulate the production of reactive oxygen species (ROS), damaging genomic stability and oxidizing membrane lipids, proteins and DNA, fundamental cellular components. In the face of this stress, some enzymes can neutralize these free radicals, such as superoxide dismutase, catalase and glutathione peroxidase [63]. The body also activates protective pathways, such as DNA repair mechanisms and the control of factors such as the nuclear factor erythroid 2–related factor 2 (Nrf2), which regulates genes related to antioxidant defense and detoxification. However, when exposure to metals exceeds the system's adaptive capacity, these mechanisms become insufficient, which can lead to the progressive accumulation of damage and, consequently, cellular dysfunction [67]. Although more detailed studies are needed on the endogenous mechanisms that combat metal toxicity, the existing literature already demonstrates the relevance of antioxidant and chelating systems in controlling damage caused by metals.

## 7 Biomaterials, implants, and microbial ecology

Implant materials are not biologically neutral, and their interaction with both host tissues and microbial communities critically determines infection outcomes. Titanium, the most commonly used orthopedic and dental implant material, is valued for its mechanical strength and biocompatibility. However, corrosion and wear processes inevitably lead to the release of titanium ions and particles, particularly under inflammatory or acidic conditions [68]. These particles induce oxidative stress, DNA damage, and epigenetic alterations in surrounding cells, creating a pro-inflammatory microenvironment that may compromise osseointegration [69]. Beyond their direct effects on host cells, metallic by-products influence microbial colonization. Released ions and surface modifications alter protein adsorption patterns on the implant, shaping the so-called “conditioning film” that governs microbial adhesion [70]. Once adhered, bacteria form biofilms that are resistant to both antibiotics and host immune defenses, turning the implant into a persistent infection reservoir [[71], [72], [73], [74], [75]]. Certain bacterial metabolites can further accelerate corrosion of implant surfaces, establishing a vicious cycle of metal release, inflammation, and biofilm stability [76]. The ecological dynamics of surgical site infections further underscore the dual role of biomaterials and microbes. Initial contamination during surgery may involve low bacterial loads that are insufficient to cause infection in healthy tissue. However, when coupled with impaired host defenses or a pro-inflammatory local environment induced by metal release, these bacteria may persist, adapt, and form biofilms [77]. Microorganisms also exhibit remarkable adaptability: exposure to oxidative stress or metal ions can select for more resilient phenotypes, including multidrug-resistant strains [78]. Interestingly, microbial colonization is not always detrimental. Commensal and probiotic organisms can modulate tissue homeostasis, compete with pathogens, and shape immune responses toward a healing phenotype [6]. This dual role emphasizes that microbial ecology on implant surfaces is not inherently pathological; rather, it depends on the balance between beneficial and harmful colonizers, as well as the local chemical and immunological milieu created by the biomaterial.

## 8 Microbes as mediators of healing and defense

### 8.1 Microbial role in tissue healing

Interest in the role of microbiota in wound healing has increased considerably, especially with regard to chronic wounds and the search for more specific treatments. Apparently, in the skin, microbiota helps in tissue recovery, affecting local immune responses and interacting directly with the cells that participate in regeneration [79]. Certain types of bacteria present in the skin microbiota, such as *Staphylococcus epidermidis*, have been linked to stimulating immune responses that help control exaggerated inflammation and boost more organized regeneration [80]. On the other hand, when there is an imbalance in the microbial composition, known as dysbiosis, opposite effects are observed, such as slower healing, increased local inflammation, and the emergence of bacterial biofilms with high resistance to antimicrobial action [79,81]. The intestinal flora also has a positive effect on tissue regeneration, relieving inflammation and allowing skin lesions to heal [82]. Various tactics that seek to influence the microbiota, such as the use of probiotics, prebiotics, or even the transplantation of commensal bacteria, may be promising alternatives in the treatment of chronic wounds, helping to restore local microbial balance and promoting tissue recovery processes [81].

#### 8.1.1 Microbial detoxification of heavy metals

**Fig. 1:**
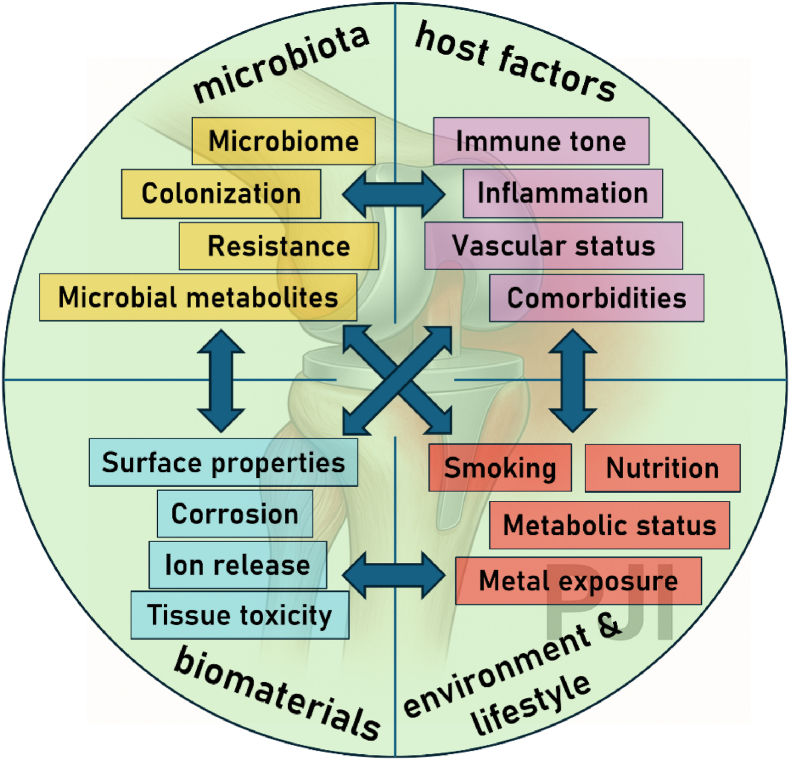
**Determinants of infection susceptibility in implant surgery: a systems perspective**. Infection risk associated with biomedical implants arises from the dynamic interplay between multiple layers of biological, material, and environmental factors. Host-related parameters such as immune tone, inflammatory state, vascular status, comorbidities, metabolic status, nutrition, and smoking habits shape the tissue microenvironment and influence susceptibility. Microbiota composition and activity, including microbial metabolites and colonization resistance, modulate local and systemic immune responses and impact pathogen dynamics. Biomaterial properties—notably surface structure, corrosion behavior, and ion release—affect microbial adhesion, biofilm formation, and tissue compatibility, while also contributing to metal exposure and potential tissue toxicity. Together, these interconnected factors determine whether the outcome of implant surgery is characterized by successful integration and tissue homeostasis or by microbial persistence and manifested infection.

Microbial mechanisms such as biosorption, bioaccumulation, precipitation, and redox reactions are described as strategies to reduce both the bioavailability and toxicity of heavy metals such as cadmium, lead, mercury, and arsenic [49]. *Lactobacillus* and other lactic acid bacteria, for example, use their exopolysaccharides to capture metal ions, which can mitigate cytotoxic damage in damaged environment [54]. Furthermore, the transformation of toxic metallic forms into less reactive compounds by microorganisms has also been reported in contaminated wounds, which can be considered as an alternative way to mitigate the deleterious effects of these contaminants also in tissue repair processes [49]. In this scenario, the intestinal microbiota is relevant, especially in the way it alters metals such as bismuth, affecting their distribution and how they are processed by the body [57]. In view of this, the use of probiotics with detoxifying action, together with adjustments in the local microbiota, appears as a promising way to accelerate recovery and help restore tissues in contaminated wounds and chronic injuries [58,83] (see Fig. 1).

### 8.2 Biofilms as potential detoxification systems: a hypothesis for Host–Microbe adaptation

**Fig. 2:**
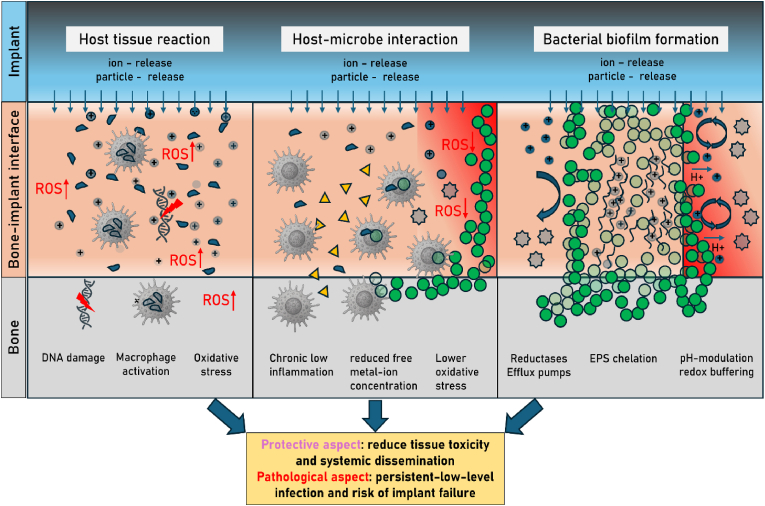
**Biofilm formation as a potential detoxification and containment response at the bone–implant interface.** Following implantation, corrosion and wear processes lead to the release of metal ions and particles, which can generate reactive oxygen species (ROS), induce oxidative stress, trigger DNA damage, and activate immune cells such as macrophages. Microorganisms colonizing the implant surface can form biofilms whose extracellular polymeric substance (EPS) matrix exhibits metal-chelating properties, pH modulation, and redox buffering, thereby reducing free metal-ion concentrations and lowering local oxidative stress. In addition, bacterial reductases and efflux pumps contribute to detoxification and metal tolerance. This biofilm-mediated containment may have a protective aspect, limiting acute tissue toxicity and systemic dissemination of metals. However, it also has a pathological aspect, supporting chronic low-grade inflammation, persistent infection, and potential implant failure. The dual nature of biofilms highlights the need to reconsider their role not solely as pathogenic entities but also as possible components of a local detoxification response.

Biofilms are traditionally described as pathogenic microbial communities that form on implant surfaces, protect bacteria from host immunity and antibiotics, and drive chronic infection. Yet biofilms are more than passive shields. Their structural and biochemical features can plausibly **buffer toxic by-products** generated after implantation, adding a detoxification dimension to host–microbe–biomaterial interactions. A defining property of biofilms is the *extracellular polymeric substance (EPS)* matrix—a heterogeneous mesh of polysaccharides, proteins, lipids, and extracellular DNA—with abundant functional groups (carboxyl, phosphate, amine, sulfhydryl, phenolic) that bind and sequester metal ions. Extensive work shows EPS can adsorb/immobilize diverse metals, thereby lowering free ion concentrations in the surrounding milieu. This chelation capacity is repeatedly demonstrated in laboratory and environmental systems and is mechanistically attributed to those EPS functional groups [[84], [85], [86], [87]]. Beyond passive binding, many biofilm-forming bacteria express active metal-detox strategies—notably *efflux pumps* (RND/CzcCBA systems, P-type ATPases, CDF transporters), enzymatic redox transformations, and metallothionein-like binding proteins—that reduce intracellular metal burden or convert ions to less reactive forms. These pathways are well documented as core components of bacterial heavy-metal resistance and are frequently upregulated in biofilms [[88], [89], [90]]. Viewed through a systemic lens, persistence of low-grade biofilms at an implant could sometimes reflect a containment equilibrium rather than outright immune failure: EPS-mediated sequestration and bacterial detox programs may limit acute oxidative stress and curb ion diffusion from corroding/wearing surfaces, even as they sustain chronic, low-level inflammation and infection risk. This *dual role*—protective containment vs. pathological persistence—argues for more nuanced inquiry [86]. This hypothesis doesn't excuse biofilms clinically; they remain central drivers of implant failure. It does, however, motivate targeted experiments (e.g., measuring local free-ion activities and ROS in vivo with/without biofilm disruption; knockouts of efflux/redox genes in implant models) and therapeutic design that separates beneficial sequestration from pathogenic resilience—for example, surfaces or adjuncts that preserve ion buffering while preventing mature biofilm architecture (Fig. 2).

## 9 Future directions and integrated perspective

A growing body of research highlights the need for integrative, host-centered strategies to reduce implant-related infections. Traditional approaches, focused on antimicrobial prophylaxis and aseptic technique, must be complemented by interventions that optimize the host terrain. Reducing systemic inflammation, enhancing nutritional status, modulating the microbiota, and minimizing exposure to immunotoxic metals should be seen as part of comprehensive perioperative care [91]. Personalized medicine may extend to implant selection, where patients with known metal sensitivities could benefit from alternative alloys or ceramic-based biomaterials that exhibit lower corrosion rates [7]. Clinical applications are beginning to emerge from this integrative perspective. Dietary modulation—through antioxidant-rich foods, omega-3 fatty acids, and micronutrient supplementation—can strengthen immune resilience and accelerate wound healing [31]. Probiotics and symbiotics, whether administered orally or applied to implant surfaces, show promise in modulating local microbial ecology and preventing pathogen overgrowth [45]. At the level of materials science, implant surfaces are being engineered to release not only antimicrobial agents but also immunomodulatory compounds that promote a balanced host response [92]. Nanotopographical modifications are another strategy, discouraging bacterial adhesion while favoring osteoblast activity [76]. Looking ahead, research priorities include clarifying the mechanisms by which metals influence microbiota composition, and how microbial metabolites affect corrosion and immune activation. Longitudinal clinical studies that integrate omics approaches—microbiome sequencing, metabolomics, and immunoprofiling—are needed to map the dynamic interplay between host, microbes, and biomaterials. Such systems-level approaches could enable predictive models of infection risk, guiding individualized prevention strategies.

## 10 Conclusion

Implant-related infections represent the convergence of microbial exposure, biomaterial properties, and host factors. While germ theory explains the central role of pathogens, a host-centered perspective underscores the importance of immune status, tissue environment, and systemic influences in shaping infection outcomes. In reality, susceptibility emerges from the dynamic interplay between host biology, microbiota, and biomaterials, with metals, oxidative stress, and immune modulation acting as critical mediators. Environmental exposures, lifestyle factors, implant design, and microbial ecology intersect in ways that either promote infection or preserve tissue homeostasis. An emerging layer of complexity is the evolving view of biofilms not only as pathogenic strongholds but also as potential participants in local detoxification. Their extracellular polymeric matrices and microbial metal-handling mechanisms can sequester and neutralize ions released from biomaterials, potentially buffering tissue from acute toxicity. This duality suggests that biofilm formation may, in part, represent an adaptive containment response — one that mitigates chemical stress at the cost of sustaining chronic low-grade inflammation and infection risk. Recognizing this functional nuance reframes biofilms as not merely a therapeutic target to eradicate, but also as a biological phenomenon to understand and strategically modulate. A future-oriented perspective must therefore embrace systems biology approaches, integrating molecular, microbial, and material science with clinical practice. Infection prevention should not be reduced to the elimination of microbes but expanded to include the cultivation of a resilient host environment, optimization of microbiota composition, and design of biomaterials that minimize toxic by-products while supporting immune balance. Integrative strategies — ranging from dietary and metabolic interventions to probiotic therapies and advanced implant surface engineering — represent the next frontier in reducing the burden of implant-associated infections. By bridging insights from immunotoxicology, microbiome research, and biomaterials science, a more comprehensive framework can emerge, guiding the development of interventions that not only prevent infection but also support long-term host–implant integration and tissue health.

## CRediT authorship contribution statement

**D.C. Coraça-Huber:** Writing – review & editing, Writing – original draft, Supervision, Project administration, Methodology, Investigation, Formal analysis, Data curation, Conceptualization. **C. Spiegel:** Writing – review & editing, Writing – original draft, Investigation, Data curation. **B.F. Moraes:** Writing – review & editing, Writing – original draft, Investigation, Formal analysis, Data curation. **R. Arora:** Writing – review & editing, Writing – original draft, Resources, Investigation, Formal analysis, Conceptualization.

## Declaration of generative AI and AI-assisted technologies in the manuscript preparation process

During the preparation of this manuscript, the authors used ChatGPT (OpenAI, USA) to assist in identifying relevant references and to improve clarity and language flow. All content generated with the assistance of this tool was subsequently reviewed, verified, and edited by the authors, who take full responsibility for the final version of the manuscript and its scientific integrity.

## Funding

This review received no external funding for its development. All authors were supported solely by their respective institutions as part of their regular employment.

## Declaration of interests

The authors declare that they have no known competing financial interests or personal relationships that could have appeared to influence the work reported in this paper.

## Data Availability

No data was used for the research described in the article.
